# The learning curve of laparoscopic splenectomy and esophagogastric devascularization for portal hypertension with 10-year follow-up

**DOI:** 10.1007/s00464-024-11017-0

**Published:** 2024-07-24

**Authors:** Dong Wang, Xiao Chen, Ling Lv, Tao Yang, Bo Huang, Yanlong Cao, Yong Zhang, Jianguo Lu, Jikai Yin

**Affiliations:** 1 Department of General Surgery, Tangdu Hospital, Fourth Military Medical University, 569 Xin Si Road, Xi’an, 710038 China; 2 Department of Disease Prevention and Health Care, Tangdu Hospital, Fourth Military Medical University, Xi’an, 710038 China

**Keywords:** Laparoscopic splenectomy, Esophagogastric devascularization, Learning curve, Cumulative summation, Portal hypertension

## Abstract

**Introduction and objectives:**

Laparoscopic splenectomy and esophagogastric devascularization (LSED) are minimally invasive, effective, and safe in treating esophageal-fundic variceal bleeding with portal hypertension (PHT). The study aimed to assess the learning curve of LSED by cumulative summation (CUSUM) analysis. The 10-year follow-up data for LSED and open surgery were also examined.

**Patients and methods:**

Five hundred and ninety-four patients were retrospectively analyzed. Operation time, intraoperative blood loss, open operation conversion, and postoperative complications were selected as the evaluation indicators of surgical ability. The learning curve of LESD was assessed by the CUSUM approach. Patient features, perioperative indices, and 10-year follow-up data were examined.

**Results:**

Totally 236 patients underwent open surgery, and 358 underwent LSED. Patient characteristics were similar between groups. The LSED patients experienced less intraoperative blood loss, fewer complications, and faster recovery compared to the open surgery cohort. The learning curve of LESD was maximal for a case number of 50. Preoperative general characteristics were comparable for both stages. But the skilled stage had decreased operation time, reduced blood loss, less postoperative complications, and better recovery compared to the learning stage. The LSED group had higher recurrent hemorrhage-free survival rate and increased overall survival in comparison with cases administered open surgery in the 10-year follow-up. Free-liver cancer rates were similar between two groups.

**Conclusions:**

About 50 cases are needed to master the LSED procedure. Compared to open surgery, LSED is a safer, feasible, and safe procedure for PHT patients, correlating with decreased rebleeding rate and better overall survival.

Liver cirrhosis, frequently resulting from hepatitis B and C infections, is highly prevalent in Asian nations and is acknowledged as the predominant cause of decompensated liver function, portal hypertension (PHT), and hepatocarcinoma. Esophagogastric varices bleeding (EVB) and splenomegaly combined with hypersplenism are the most common complications of PHT. Endoscopic treatment is regarded as the first-line therapy for EVB; however, bleeding recurrence rates still remain high [1–3]. Transjugular intrahepatic portosystemic shunt (TIPS) effectively decreases EVB and rebleeding, but decreased liver function and hepatic encephalopathy after TIPS cannot be ignored. Neither endoscopic treatment nor TIPS treatment effectively solves hypersplenism and splenomegaly. Several studies have shown that splenomegaly induces the secretion of cytokines, deteriorating liver function and increasing the incidence of hepatocellular carcinoma [4–8]. Splenectomy with esophagogastric devascularization (SED) can simultaneously solve both issues of EVB and splenomegaly, thereby widely applicated in China and Japan. However, the substantial trauma associated with traditional open SED (OSED) surgery has confined its application, especially in western countries where acceptance remains limited.

Laparoscopic surgery is a less invasive procedure compared with the traditional open surgery. Although laparoscopic splenectomy and esophagogastric devascularization (LSED) for PHT treatment is considered a high-risk operation, conversion from traditional open surgery to laparoscopic surgery has been gradually performed with advancements in laparoscopic techniques and equipment. The LSED procedure was firstly performed in 2011 in our department and has now become the initial procedure for PHT. Though several studies [9–11] have investigated the learning curve for pure laparoscopic splenectomy in non-portal hypertensive patients, to our knowledge, no study has reported the learning curve for LSED in PHT. This study aimed to assess the feasibility, safety, and efficiency of the LSED relative to traditional OSED, and to explore the learning curve of LSED by cumulative summation (CUSUM) analysis. The benefits of LSED in decreasing esophagogastric varices rebleeding and extending overall survival in PHT patients were also determined.

## Meterials and methods

### Patients

Retrospective analysis was conducted on 594 PHT patients with liver cirrhosis who underwent either OSED or LSED at the Department of general surgery, Tangdu Hospital, affiliated by Fourth Military University, from January 2005 to November 2020. Two hundred and thirty-six patients underwent the OSED procedure between January 2005 and December 2010. Totally 358 patients from January 2011 underwent the LSED procedure, which has become the initial surgical option for PHT patients. The preoperative characteristics of the patients are summarized in Table 1. This study followed the Declaration of Helsinki and had approval from the Research Ethics Committee of Tangdu Hospital (202011-32). Each patient provided signed informed consent. Samples were anonymous as required by ethical and legal standards.

**Table 1:** The baseline patient data between OSED group and LSED group

Factors	OSED (*n* = 236)	LSED (*n* = 358)	*p*
Age (years)	44.59 ± 11.73	45.42 ± 10.59	0.375
Sex			0.120
Male	147 (62.3%)	200 (55.9%)	
Female	89 (37.7%)	158 (44.1%)	
BMI (kg/m^2^)	22.1 ± 3.29	22.52 ± 2.8	0.093
Causes of cirrhosis			0.830
HBV	159 (67.4%)	249 (69.6%)	
HCV	31 (13.1%)	42 (11.7%)	
Others	46 (19.5%)	67 (18.7%)	
MELD	9.89 ± 2.35	9.63 ± 2.3	0.179
Child–Pugh score	6 (5, 7)	6 (5, 7)	0.096
Child–Pugh classification			0.057
A	152 (64.4%)	257 (71.8%)	
B	84 (35.6%)	101 (28.2%)	
Variceal hemorrhage history			0.183
Negative	87 (36.9%)	112 (31.3%)	
Positive	149 (63.1%)	246 (68.7%)	
Varicose vein diameter (mm)			0.142
≥ 5	182 (77.1%)	294 (82.1%)	
< 5	54 (22.9%)	64 (17.9%)	
Red color sign			0.068
Negative	49 (20.8%)	98 (27.4%)	
Positive	187 (79.2%)	260 (72.6%)	
RBC (× 10^12^/L)	3.52 ± 0.83	3.46 ± 0.88	0.442
WBC (× 10^9^/L)	3.13 ± 1.24	3.06 ± 1.21	0.473
ALT (U/L)	27 (20, 41)	28 (21, 38.25)	0.731
AST (U/L)	37 (28, 51)	29 (21.75, 40)	**0.000**
ALB (g/L)	37.7 ± 4.24	37.83 ± 4.1	0.702
TBIL (umol/L)	25.13 ± 12.86	23.21 ± 10.36	0.055
PLT (× 10^9^/L)	49.15 ± 29.17	53.5 ± 40.43	0.155
PT (s)	14.03 ± 1.92	13.74 ± 1.77	0.067
Spleen			
Length (cm)	17.74 ± 10.33	17.62 ± 2.87	0.834
Thickness (cm)	6.09 ± 1.3	6.13 ± 1.27	0.750
Portal vein diameter (cm)	1.47 ± 0.25	1.46 ± 0.23	0.732
Portal vein velocity (cm/s)	16.79 ± 5.85	17.16 ± 5.24	0.421
Portal vein flow (mL/min)	1753.91 ± 1092.41	1715.53 ± 762.32	0.616

Bold value indicates the statistical difference

The inclusion criteria for surgery were (1) a confirmed history of EVB secondary to cirrhotic PHT; (2) a pathological diagnosis of congestive splenomegaly combined with severe hypersplenism (white blood cell count < 2.0 × 10^9^/L and platelet count < 50 × 10^9^/L); and (3) no history but a high risk of EVB (endoscopy showing grade III esophageal or blue varices, or cherry red spots of bleeding varices, combined with severe hypersplenism, and high hepatic venous pressure gradient (HVPG) > 12 mmHg). Patients with Child–Pugh C class, > 70 years old, or with hepatocellular carcinoma were excluded. Detailed clinical and biochemical evaluations and enhanced abdominal CT were performed preoperatively. Postoperative mortality was defined as death occurring within 90 days following the surgery.

### Surgical procedure

**Fig. 1:**
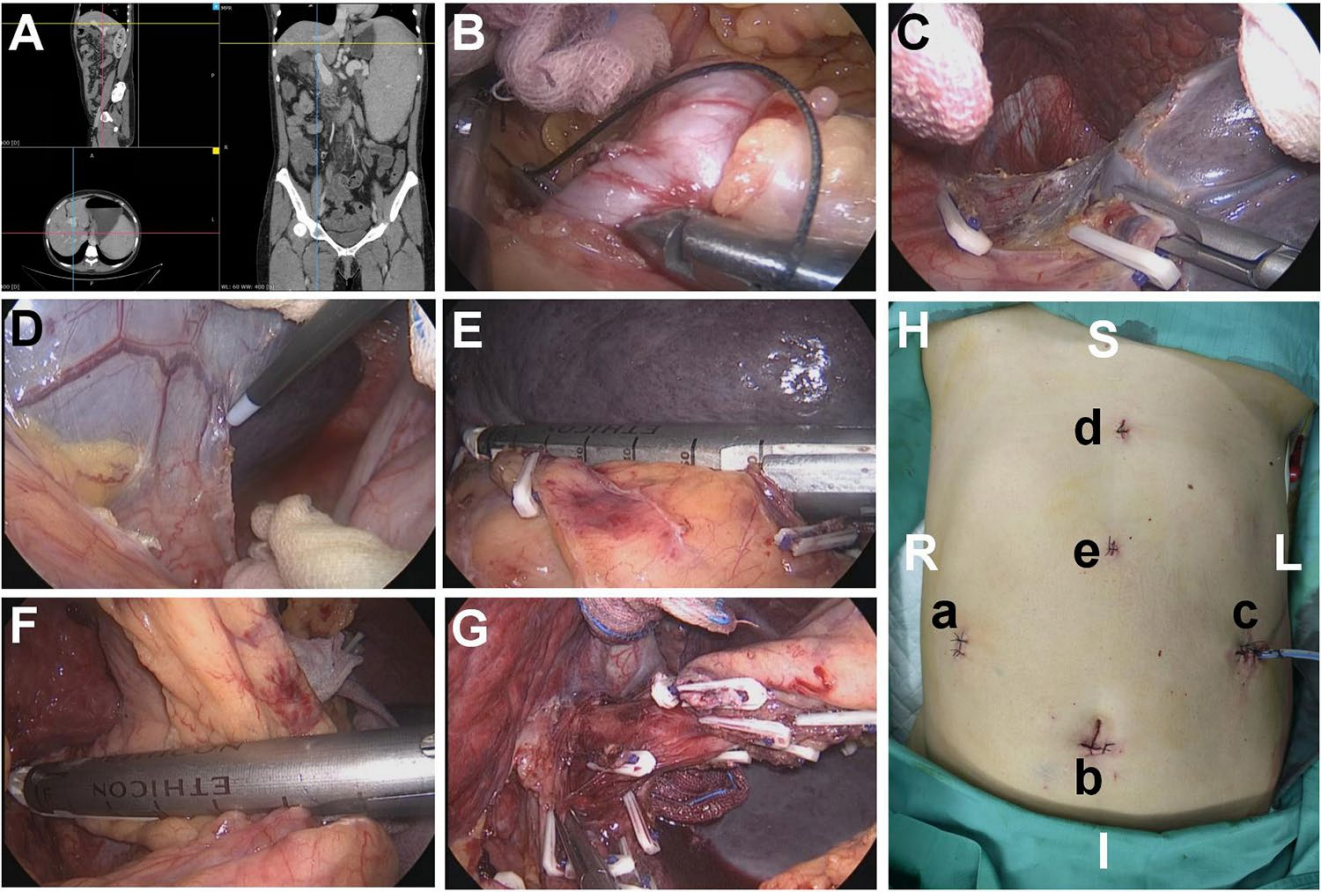
Evaluation and main steps in laparoscopic splenectomy and esophagogastric devascularization procedure (showed with the same patient). **A** Preoperative abdominal enhanced CT evaluation; **B** the splenic artery separation and ligation; **C **short gastric vessels dissection by absorbable hemostatic clamps; **D** the dorsal splenic ligament separation; **E** splenic hilar pedicles transection with the Endo GIA™ stapler; **F** the left gastric vein transection with the Endo GIA™ stapler; **G** esophagogastric devascularization by absorbable hemostatic clamps; **H** laparoscopic surgery completion with an abdominal drain. Trocar locations: laparoscopic observation hole (a); Main operative holes (b, c); Assistant operative holes (d, e). *S* superior, *I* inferior, *R* right, *L* left

In the LSED group, patients were placed supine with the left flank elevated at a 30° angle. Five operative ports were used as reported previously [12–14]. The specific surgical steps are shown in Fig. 1. Briefly, after insertion of a 12-mm laparoscopic trocar beneath the umbilicus, four additional trocars were inserted, including at the crossover point of the right axillary midline and navel horizontal line (12 mm trocar), the level of the spleen’s lower pole (12 mm trocar), the point along the ventrimeson and 3 cm below the xiphoid process along the ventrimeson and (5 mm trocar), and the crossover point of the ventrimeson and last rib margin (5 mm trocar). Electrocautery, the LigaSure™ vessel-sealing system (Medtronic, USA), absorbable hemostatic clamps, and the Endo GIA™ stapler (Medtronic, USA) were utilized for vessel disruption. Following dissection of gastrocolic and gastrosplenic ligaments, the splenic artery was separated and ligated above the superior border of the pancreas. The volume of the enlarged spleen decreased with splenic artery ligation, thereby creating more operative field, facilitating spleen dissection, and minimizing accidental bleeding. Enlarged short gastric vessels are deepen in the superior position of the gastrosplenic ligament; therefore, dissection should be meticulously executed in close proximity to the spleen to prevent bleeding and avoid damaging the gastric serosa. The short gastric vessels dissection will effectively sever the blood flow from the enlarged spleen to the gastric fundus. Similarly, the splenorenal and splenocolic ligaments were separated. The splenic hilum underwent transection with an Endo GIA™ stapler (60–2.5 mm) to complete the splenectomy. The root of the left gastric vein was then transected with the same type of stapler, followed by resection of soft tissues and varices along the greater and lesser curvature of the stomach, as well as a 5-cm segment of the lower esophagus. Throughout the devascularization, the posterior gastric veins, as well as the gastric, esophageal, and high esophageal branches of left gastric vein, and potentially present left inferior phrenic veins were disconnected. The process effectively interrupts the majority of the abnormal portosystemic collaterals connecting the portal vein to the esophagogastric fundus. The spleen was fragmented in the specimen bag in the abdomen and removed from the left lower quadrant-extended incision. An abdominal drain was routinely placed in the left upper abdomen in all patients.

Traditional OSED was performed with patients employing a left subcostal "L"-shaped incision. This procedure was comparable to laparoscopic surgery, but surgical techniques including clamp, transection, ligation, and suture were performed in a conventional way.

### Operative outcomes and complications

Surgical outcomes and postoperative complications are shown in Table 2. Postoperative morbidity assessment utilized the Clavien-Dindo classification [15], and grade ≥ III complications were considered serious complications.

**Table 2:** Perioperative data and complications between OSED group and LSED group

Factors	OSED (*n* = 236)	LSED (*n* = 358)	*p*
Perioperative data			
Operation time (min)	230 (180, 290)	280 (240, 340)	**0.000**
Blood loss (mL)	700 (400, 1300)	500 (200, 1000)	**0.000**
Duration of getting out of bed (days)	2 (2, 3)	2 (2, 2)	**0.000**
Abdominal drain (days)	5 (5, 6)	5 (3, 6)	**0.000**
Postoperative hospital stays (days)	10 (9, 13)	8 (7, 9)	**0.000**
Open operation conversion	N/A	30 (8%)	N/A
Perioperative complications			
Intra-abdominal bleeding	17 (7.2%)	12 (3.4%)	**0.033**
Pancreatic fistula	1 (0.4%)	2 (0.6%)	0.713
Encephalopathy	0	0	–
Severe ascites	11 (4.7%)	6 (1.7%)	**0.033**
Intra-abdominal infection	10 (4.2%)	5 (1.4%)	**0.031**
Reoperation after LSD	8 (3.4%)	10 (2.8%)	0.678
Clavien-Dindo grade	31 (12.9%)	28 (7.8%)	**0.039**
IIIA	8	5	
IIIB	8	10	
IVA	7	7	
IVB	4	5	
V	4	1	

Bold values indicate the statistical difference

Grade IIIA: OSED group (8), 8 patients received gastroscopic hemostasis; LSED group (5), 5 patients received gastroscopic hemostasis

Grade IIIB: OSED group (8), 8 patients underwent re-laparotomy for intra-abdominal bleeding; LSED group (10), 9 patients underwent re-laparotomy for intra-abdominal bleeding and 1 patient underwent re-laparotomy for gastric fistula repair

Grade IVA: OSED group (7), 1 patient needed ICU management due to the hypotension and shock, 3 patients needed ICU management due to the renal failure and 3 patients due to the pulmonary embolism; LSED group (7), 2 patients needed ICU management due to the hypotension and shock, 2 patients due to the liver failure, and 3 patients due to the pulmonary embolism

Grade IVB: OSED group (4), 4 patient needed ICU management due to the hepatorenal syndrome; LSED group (5), 5 patients needed ICU management due to the hepatorenal syndrome

Grade V: OSED group (4), 1 patient died of septic shock and 3 patients died of intra-abdominal bleeding within 1 months; LSED group (1), 1 patient died of intra-abdominal bleeding post-discharge within 1 months

### Follow-up

Routine follow-up analysis was carried out for each patient. Long-term follow-up was conducted by telephone or hospital visits. The end of the follow-up period was March 10, 2023, and this work only reports follow-up data for the first 10 years after operation. Primary endpoints encompassed overall survival (OS, time elapsed between surgery and death from any cause) and recurrent hemorrhage-free survival (RHFS, time elapsed between surgery and the initial postsurgical EVB). In addition, one further endpoint was selected, i.e., hepatocellular carcinoma-free survival (HCFS, time elapsed between surgery and the initial postoperative definitive diagnosis of hepatocellular carcinoma).

### Cumulative sum method and learning curve analysis

CUSUM analysis was applied to quantitatively estimate the learning curve for the LSED procedure. In order to perform multidimensional CUSUM analysis, operation time, intraoperative blood loss, open operation conversion, and postoperative complications were set as evaluation indicators of surgical ability. For each case, the four evaluation indicators were quantitated as values *a*_1 to_ *a*_4_, respectively, based on the following formula: *a* = *X*_i_ − *X*_0_, where *X*_i_ and *X*_0_ are the individual attempt and reference/target value for the procedure, with *X*_i_ = 1 reflecting an attempt below standard (median intraoperative blood loss and operation time) or failure (conversion to open surgery and postoperative complications) and *X*_i_ = 0 reflecting an up-to-standard attempt (median intraoperative blood loss and operation time) or success (no conversion to open surgery or postoperative complications). The targets for median intraoperative blood loss and operation time were 500 mL and 280 min, respectively. Target rates for conversion to open surgery and postoperative complications were set at 8% and 8%, respectively. *X*_0_ values for a_1_ to a_4_ were 0.42, 0.44, 0.05, and 0.2, respectively. Therefore, surgical competence was quantitated as *S* = *a*_1_ + *a*_2_ + *a*_3_ + *a*_4_ for each patient. Scores were added, and a graph was generated with the equation: CUSUM = ∑*S*_i_.

### Statistical analysis

SPSS 26.0 and R 4.1.3 were employed for data analysis. Normally and skewedly distributed continuous variates were reported as mean ± standard deviation and median (interquartile range), respectively, and compared by the *t* test and the Mann–Whitney *U* test, respectively. Categorical variates were reported as n (%) and compared by the Fisher’s exact test or the chi-squared test. *K*–*M* curve analysis was carried out, with comparisons using the log-rank test. The learning curve was fitted by the R software. *p* values < 0.05 (two-tailed) reflected statistical significance.

## Results

### Patient features

Table 1 summarizes the baseline patient features. There were no significant differences observed between groups in terms of sex, age, body mass index, causes of cirrhosis, history of EVB, varicose vein diameter, red color sign, and Child–Pugh score/classification. Similarly, there were no statistical differences observed in liver biochemical indices (with the exception of glutamic oxaloacetic transaminase), spleen dimensions (length and thickness), or portal vein hemodynamics (including diameter, velocity, and flow).

### Perioperative outcomes and postoperative complications

Patients who underwent LSED experienced longer operative time but with significantly less intraoperative blood loss compared with those received OSED (both *p* = 0.000). Following LSED, we observed a shortened median duration of abdominal drainage and a quicker postoperative discharge compared to OSED (both *p* = 0.000). Thirty out of 358 patients administered LSED required conversion to open surgery due to intraoperative abdominal bleeding and difficult tissue separation.

More patients suffered from intra-abdominal bleeding in the OSED group compared with the LSED group (*p* = 0.033). Intra-abdominal infection and severe ascites had starkly higher rates in the OSED group compared with cases administered LSED (*p* = 0.031 and *p* = 0.033, respectively). More complications above Clavien-Dindo grade III were detected in the OSED group compared to the LSED group (*p* = 0.039). No significant difference in pancreatic fistula and reoperation rate was found between the two groups. The data are summarized in Table 2.

### Learning curve based on CUSUM analysis

**Fig. 2:**
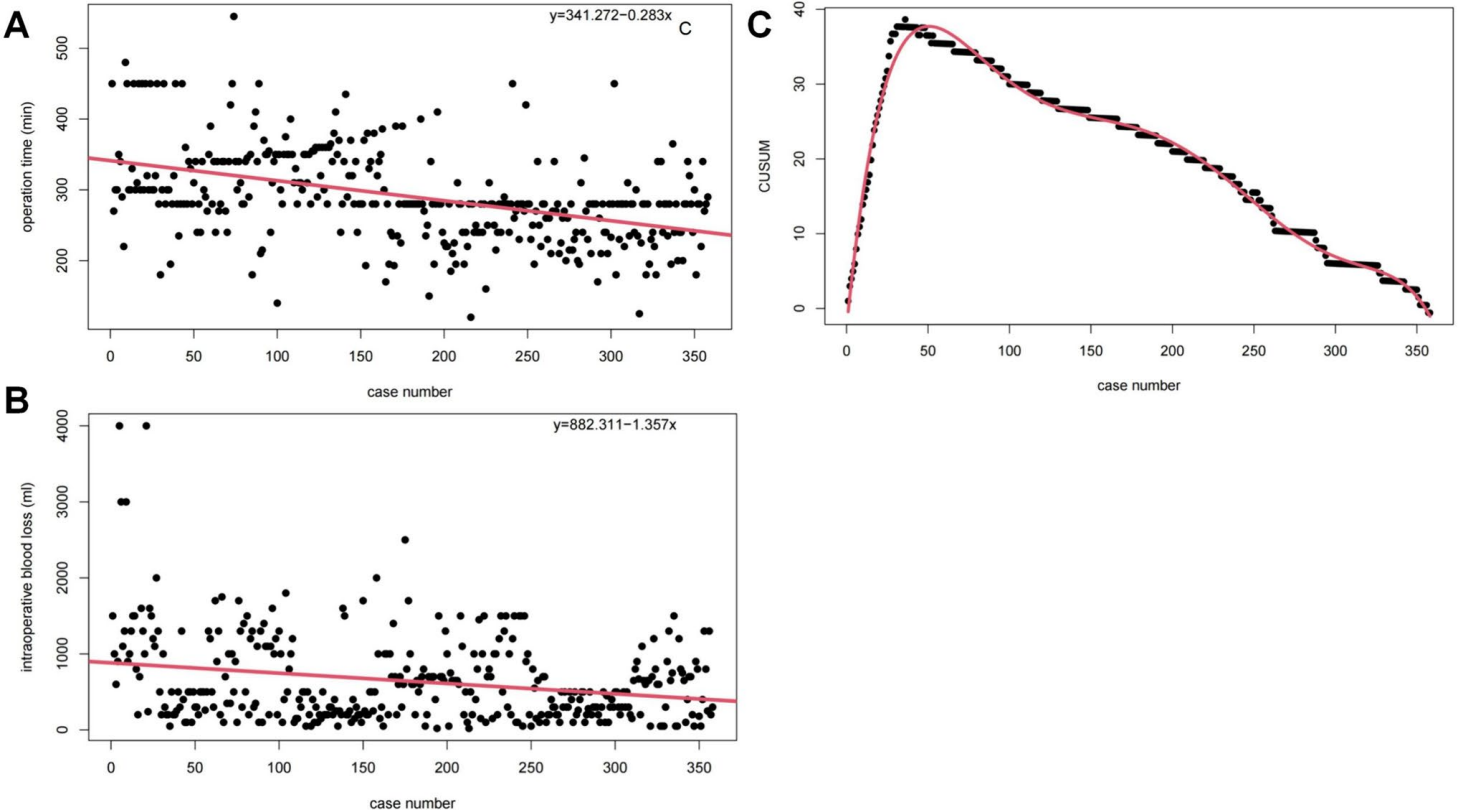
**A** The scatter chart of operation time with trend line; **B** the scatter chart of intraoperative blood loss with trend line; **C** CUSUM for learning curve reached the maximum in No. 50 case, revealed competency after 50 cases

The scatter plots of operation time and intraoperative blood loss both revealed an overall downward trend (Fig. 2A, B). The CUSUM analysis for the learning curve indicated the maximum occurring after the 50th case, and then gradually declined (Fig. 2C). The learning curve’s optimal fit was described by a fourth-order polynomial equation: 2.559 + 2.163 × (Case number) − 4.145 × 10^−2^ × (Case number)^2^ + 3.556 × 10^−4^ × (Case number)^3^ − 1.547 × 10^−6^ × (Case number)^4^ + 3.298 × 10^−9^ × (Case number)^5^ − 2.738 × 10^−12^ × (Case number)^6^, with an *R*-value of 0.992. Therefore, the 50th case was identified as a cut-off point in the learning curve’s analysis, demarcating the initial phase (comprising the first 50 cases) from the advanced phase (cases beyond the 308th). Preoperative general characteristics between these two phases revealed no statistically significant differences (*p* > 0.05), as detailed in Table 3. Perioperative data and operation complications showed significant improvements in the advanced stage compared to the initial stage (*p* < 0.05). Better postoperative liver function recovery was detected in patients of the advanced stage compared with those in the initial stage (*p* < 0.05, Table 4).

**Table 3:** The baseline patient data between the first and latter stage in LSED group

Characteristics	First stage (*n* = 50)	Latter stage (*n* = 308)	*p*
Age (years)	44.22 ± 11.19	45.61 ± 10.5	0.390
Sex			0.062
Male	16 (32.0%)	142 (46.1%)	
Female	34 (68.0%)	166 (53.9%)	
BMI (kg/m^2^)	22.42 ± 2.53	22.54 ± 2.85	0.786
Causes of cirrhosis			0.228
HBV	39 (78.0%)	210 (68.2%)	
HCV	6 (12.0%)	36 (11.7%)	
Others	5 (10.0%)	62 (20.1%)	
MELD	10.09 ± 2.06	9.53 ± 2.32	0.108
Child–Pugh score	6.5 (5, 7)	6 (5, 6)	0.105
Child–Pugh classification			0.187
A	32 (64.0%)	225 (73.1%)	
B	18 (36.0%)	83 (26.9%)	
Variceal hemorrhage history			0.438
Negative	18 (36.0%)	94 (30.5%)	
Positive	32 (64.0%)	214 (69.5%)	
Varicose vein diameter (mm)			0.709
≥ 5	42 (84.0%)	252 (81.8%)	
< 5	8 (16.0%)	56 (18.2%)	
Red color sign			0.109
Negative	9 (18.0%)	89 (28.9%)	
Positive	41 (82.0%)	219 (71.1%)	
RBC (× 10^12^/L)	3.59 ± 0.83	3.44 ± 0.88	0.259
WBC (× 10^9^/L)	3.08 ± 1.29	3.05 ± 1.19	0.909
ALT (U/L)	30.5 (21, 40.25)	28 (21, 38)	0.431
AST (U/L)	34 (23.75, 43)	28 (21, 38.75)	0.116
ALB (g/L)	38.21 ± 3.84	37.77 ± 4.14	0.484
TBIL (umol/L)	24.63 ± 11.82	22.98 ± 10.1	0.296
PLT (× 10^9^/L)	49.5 ± 22.89	54.15 ± 42.6	0.452
PT (s)	13.89 ± 1.78	13.55 ± 1.62	0.175
Spleen length (cm)	17.16 ± 2.98	17.7 ± 2.85	0.220
Spleen thickness (cm)	6.19 ± 2.21	6.12 ± 1.04	0.709
Portal vein diameter (cm)	1.44 ± 0.22	1.46 ± 0.23	0.542
Portal vein velocity (cm/s)	18.37 ± 7.04	16.97 ± 4.87	0.186
Portal vein flow (mL/min)	1703.24 ± 850.17	1723.4 ± 743.72	0.863

**Table 4:** perioperative data and complications in two stages of LSED patients

Characteristics	First stage (*n* = 50)	Latter stage (*n* = 308)	*p*
Operation time (min)	300 (300, 450)	280 (240, 320)	** < 0.01**
Blood loss (mL)	1000 (700, 1500)	500 (200, 1000)	** < 0.01**
Duration of getting out of bed (days)	4 (2, 4)	2 (2, 3)	**0.010**
Abdominal drain (days)	6 (5, 7)	4 (3, 5)	** < 0.01**
Postoperative hospital stays (days)	10 (8, 11)	8 (7, 9)	** < 0.01**
Open operation conversion			** < 0.01**
Yes	11 (22.0%)	19 (6.2%)	
No	39 (78.0%)	289 (93.8%)	
Operation complications (≥ Clavien-Dindo grade IIIA)			** < 0.01**
Yes	11 (22.0%)	18 (5.8%)	
No	39 (78.0%)	290 (94.2%)	
Postoperative biochemical indexes			
ALT (U/L)	27 (20, 42)	30 (21, 38)	0.932
AST (U/L)	33 (26, 48)	28.5 (20, 37)	** < 0.01**
ALB (g/L)	36.8 ± 4.94	37.74 ± 14.84	0.657
TBIL (umol/L)	30.70 ± 17.78	24.69 ± 10.58	** 0.024**
PLT (× 10^9^/L)	169.92 ± 88.82	241.07 ± 127.81	** < 0.01**
PT (s)	15.40 ± 2.39	13.37 ± 1.76	** < 0.01**

Bold values indicate the statistical difference

### Follow-up

**Fig. 3:**
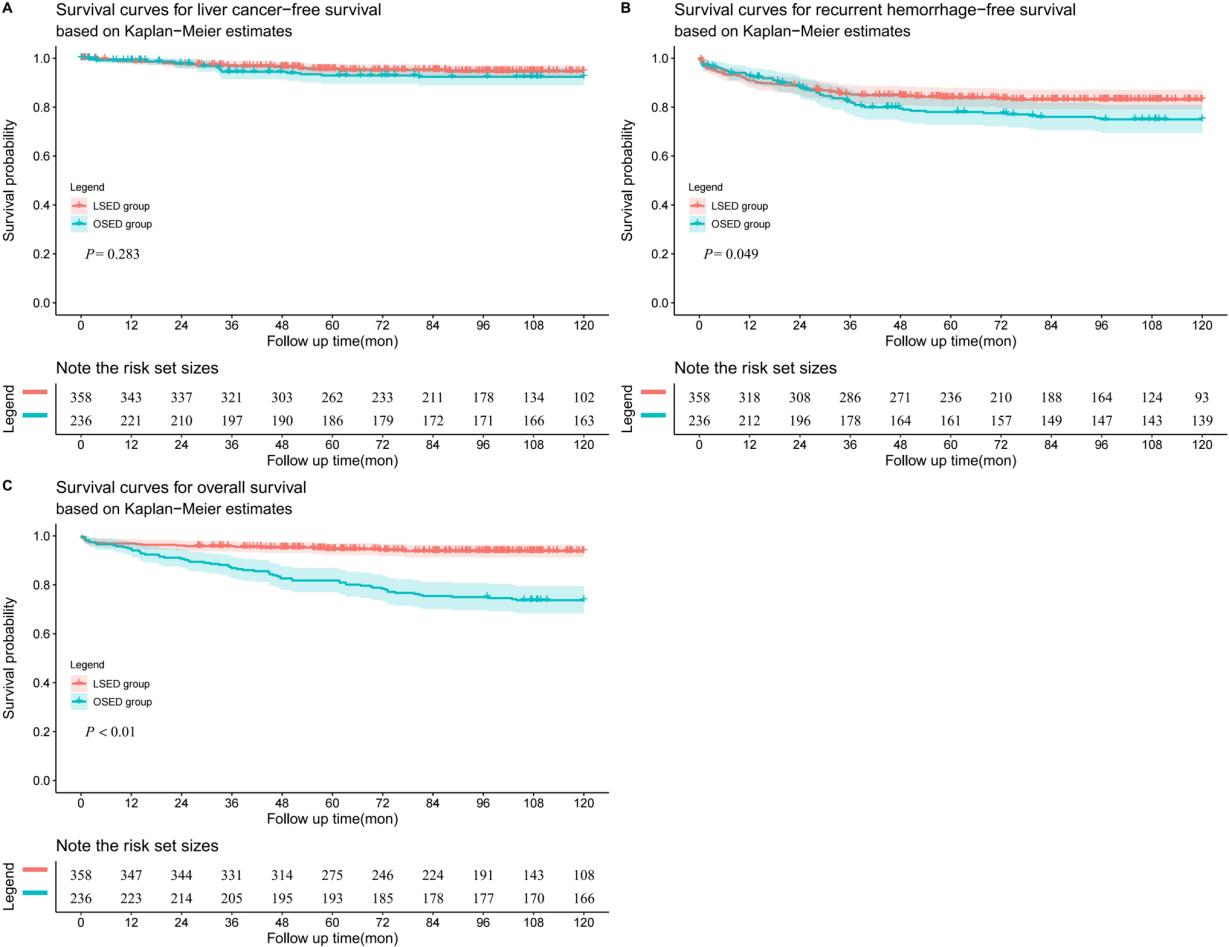
Survival curves for liver cancer-free survival (**A**), recurrent hemorrhage-free survival (**B**), and overall survival (**C**) based on Kaplan–Meier estimates

During the first 10 years of follow-up, recurrent EVB occurred in 55 patients from OSED group and in 58 patients from LSED group. Notably, the LSED group exhibited a significantly higher 10-year RHFS rate in comparison to the OSED group, with respective rates of 83.1% and 75.0% (*p* = 0.049). Sixteen cases were identified liver cancer in the OSED group and 17 cases in the LSED group. Free-liver cancer rates were similar in both groups (*p* = 0.283). During the 10-year follow-up, sixty-two patients in OSED group and 21 patients in LSED group died respectively. Markedly elevated 10-year overall survival rates were found in the LSED group compared to the OSED group (93.8% versus 73.3%, *p* < 0.01; Fig. 3).

## Discussion

Laparoscopic surgery constitutes the initial option for abdominal surgery in many medical centers. However, due to poor liver function, bleeding tendency, the presence of large abnormal varicose vessels within the abdomen, and the restricted operative space caused by massive splenomegaly, LSED is only carried out in major specialized hospitals in China. Our department has accumulated over a decade of experience in LSED, affirming its safety and effectiveness. With precisive evaluation, the LSED procedure could even be effectively and safely performed in portal hypertensive patients with massive splenomegaly [13], strongly challenging the European Society’s recommendation that huge splenomegaly is contra-indicated for laparoscopic surgery [16]. This study represents the inaugural exploration of the learning curve associated with LSED utilizing CUSUM analysis. Our findings also highlight that LSED not only successfully diminishes the 10-year rebleeding incidence but also notably enhances 10-year overall patient survival rates compared with OSED, further reinforcing the value of LSED in this complex patient cohort.

Despite longer operation time in LSED patients, reduced intraoperative bleeding, faster postoperative recovery, and a lower incidence of postoperative complications were found in the LSED group compared to those who underwent OSED. Compared with pure laparoscopic splenectomy for non-portal hypertensive cases, LSED for PHT necessitates advanced surgical skills and impeccable teamwork due to the limited operative field and heightened risk of operative hemorrhage. Additionally, extensive fibrous adhesions, particularly on the surfaces of massive splenomegaly, and previous endoscopic interventions for EVB, which can lead to inflammatory changes around the esophagus and gastric fundus, further augment the complexity and bleeding risk during LSED. The precision of laparoscopic techniques minimizes intraoperative blood loss. With the superior visualization and magnification offered by laparoscopy, meticulous dissection of structures such as the splenorenal ligament, short gastric vein, and gastroesophageal varicose veins, which are deeply situated and prone to bleeding, are much easier to process compared to the traditional open surgery. Accurate anatomical level and fine anatomy are beneficial to reducing unnecessary vascular injury during the operation, decreasing intraoperative blood loss. Due to compromised liver function and unrestored platelet function and quantity, patients with PHT are more susceptible to postoperative bleeding than other patients. Minimizing intraoperative bleeding is pivotal to preserving liver function and maintaining adequate platelet count and quality, thereby reducing the risk of postoperative bleeding. Therefore, accurate anatomical operation, complete hemostasis, and reduced intraoperative blood loss all contributed to the decrease of postoperative intra-abdominal bleeding and liver recovery in the LSED group. Abdominal hemorrhage could be stopped in most patients after transfusion of fresh frozen plasma, coagulation factors, and red blood cells when necessary. However, in our clinical experience, cases with rapid pure bleeding above over 100 mL per hour for 4 h postoperatively or more than 200 mL over 2 consecutive hours coupled with obvious hemoglobin decline and altered general vital signs need emergency reoperation. Through laparoscopic exploration, abdominal hemorrhage mostly occurs in the retroperitoneum posterior to the spleen or at the root of the left gastric vein. Careful dissection along the spleno-retroperitoneal plane and direct suturing for vessel injuries, instead of relying solely on energy devices, ensures effective hemostasis. Moreover, trocar-site bleeding, an easily overlooked complication, can result in postoperative abdominal hemorrhage. The dilated abdominal wall veins, especially the reopened umbilical vein beneath the umbilicus, were easily injured during trocar injection. Complete peritoneum suture at the trocar site for integrity restoration is a requirement for avoiding postoperative bleeding. Several studies have reported minimal invasive surgery effectively decreases postoperative infection compared with open surgery [17, 18]. In parallel, our findings also suggest that LSED is associated with a lower rate of postoperative infections. According to the clinical data collected in this article, postoperative abdominal infections are potentially arise from surgical procedures, spontaneous bacterial peritonitis, pancreatic fistulas, gastrointestinal injuries(the serous membrane on the greater curvature of the stomach injuries during the splenectomy). Overall, both precise operation and complete homeostasis contribute to decreased postoperative complications and faster recovery in the LSED group.

Currently, a limited number of articles have investigated the comparative safety of LSED versus open SED [19, 20], yet none have documented the learning curve specific to LSED. Our department initiated LSED procedures in 2011, at a time when laparoscopic splenectomy reports were scarce, with no established protocols for LSED execution. Consequently, we systematically refined every aspect, including trocar placement, anatomical demarcations, and surgical team collaboration, culminating in a standardized procedural operation process. CUSUM analysis serves as a standard quantitative methodology for evaluating surgical learning curves [21]. In this study, operation time, intraoperative bleeding, conversion to open surgery, and postoperative complications were selected as factors for CUSUM analysis. As the CUSUM curve apex indicated, operation times and blood loss progressively diminished alongside a rising caseload, dividing our study into two phases: the initial 1–50 cases and the subsequent 51–308 cases. With a learning and acclamation period, operators gain familiarity with the laparoscopic equipment and surgical steps. At the same time, with continuous optimization of surgical procedures and the cooperative involvement of other experts, a more stable learning curve is gradually achieved. General patient data were similar in both stages. However, marked improvements were observed in the later phase concerning operation time, blood loss, hospitalization duration, abdominal drain days, postoperative hospital stays, open operation conversion, and operation complications. Of note, as detailed in Table 4, the median intraoperative blood loss was slashed from 1000 to 500 mL, marking the most profound transformation between the phases. Advancements in understanding laparoscopic anatomy, adoption of sophisticated endoscopic suturing techniques, and heightened surgical team cooperation in the later phase collectively contributed to this substantial decrease in blood loss. This reduction, in turn, expedited operations, lowered conversion rates to open surgery, and curtailed postoperative complications, such as hemorrhage, by minimizing inadvertent vascular trauma and employing dependable hemostatic strategies.

Reduced recurrence of EVB and increased overall survival are important indicators of treatment success in PHT [22–24]. Nowadays, all other treatments, except for liver transplantation, are symptomatic and cannot fundamentally alleviate PHT. The reported 10-year survival rate of PHT patients administered SED treatment is about 70.7% [25], which is similar to the 73.3% reported in this article. And splenectomy was reported be beneficial for reducing the incidence of liver cancer [26]. Although both groups had comparable incidence rates of postoperative liver cancer, LSED resulted in markedly elevated 10-year RHFS versus OSED, which may contribute to the improved overall survival observed postoperatively. EVB is a critical life-threatening in PHT patients. Even with successful hemostasis, hemorrhage greatly deteriorates liver function and shortens overall survival. Therefore, decreasing the initial bleeding and rebleeding remains a paramount concern in relevant clinical guidelines and expert consensus. The reduced rebleeding rate after LSED may be attributed to a more thorough and precise esophagogastric devascularization process. Usually, esophagogastric devascularization contains the disconnection of the gastric coronary vein (including gastric, esophageal, and high esophageal branches), alongside the short gastric, posterior gastric, and left inferior phrenic veins. However, due to the potential limitation and operational difficulty of open surgery, many varicose veins, especially the high esophageal branch, are unintentional omitted, resulting in incomplete devascularization. Conversely, laparoscopic surgery permits the separation of the esophageal celiac segment by about 5–10 cm, facilitating comprehensive exposure and dissection of the varicose veins around the gastric fundus and the esophagus. In addition, clarifying the source of the varicose vein by accurate preoperative CT imaging is also helpful for precise and individualized devascularization [27], thereby contributing to the improved outcomes observed with LSED.

The limitation of the study was a single-center investigation. There might be a certain deviations difference between surgeons, their learning curve, and surgical devices available for use due to the large time span of the study.

## Conclusion

As a new minimally invasive surgery for PHT, the LSED surgery requires skills for operating laparoscopic instruments, further understanding of anatomical structures from the perspective of laparoscopy and the collaboration of the whole surgical team. Though the learning curve is steep, after overcoming the learning curve, LSED demonstrates numerous benefits, including minimized invasiveness, operational efficiency, and rapid recovery. Furthermore, LSED appears to offer decreased risk of rebleeding and better overall survival compared with the traditional open surgery, thereby reinforcing its value as a viable and advantageous alternative.
